# Ultra-High Molecular Weight Polyethylene: Influence of the Chemical, Physical and Mechanical Properties on the Wear Behavior. A Review

**DOI:** 10.3390/ma10070791

**Published:** 2017-07-13

**Authors:** Pierangiola Bracco, Anuj Bellare, Alessandro Bistolfi, Saverio Affatato

**Affiliations:** 1 Department of Chemistry and NIS (Nanostructured Interfaces and Surfaces) Center, University of Torino, 10125 Torino, Italy; 2 Department of Orthopedic Surgery, Brigham and Women’s Hospital, Harvard Medical School, Boston, MA 02115, USA; anuj@alum.mit.edu; 3 CTO Hospital, Città della salute e della Scienza, 10126 Torino, Italy; abistolfi@cittadellasalute.to.it; 4 Medical Technology Laboratory, Rizzoli Orthopaedic Institute, 40136 Bologna, Italy; affatato@tecno.ior.it

**Keywords:** UHMWPE, Ultra-high molecular weight polyethylene, oxidation, degradation, gamma radiation, crosslinking, Vitamin E, mechanical properties, wear

## Abstract

Ultra-high molecular weight polyethylene (UHMWPE) is the most common bearing material in total joint arthroplasty due to its unique combination of superior mechanical properties and wear resistance over other polymers. A great deal of research in recent decades has focused on further improving its performances, in order to provide durable implants in young and active patients. From “historical”, gamma-air sterilized polyethylenes, to the so-called first and second generation of highly crosslinked materials, a variety of different formulations have progressively appeared in the market. This paper reviews the structure–properties relationship of these materials, with a particular emphasis on the in vitro and in vivo wear performances, through an analysis of the existing literature.

## 1. Introduction

Ultra-high molecular weight polyethylene (UHMWPE) has been used as a bearing material in total joint arthroplasty for more than 50 years now. The idea to replace degraded cartilage with a polymer liner dates back to the late 1950s. At that time, Sir John Charnley chose polytetrafluoroethylene (PTFE), a low friction polymer, as the bearing material to replace the natural acetabulum, articulating against a metallic femoral head, for hip replacement. Due to the unacceptably low wear resistance of PTFE though, the first “low friction arthroplasties” dramatically failed after few years of implantation. In 1962, UHMWPE, a similarly low-friction, but much more wear resistant polymer, replaced PTFE in Charnley’s hip arthroplasty, with remarkably better performances. From then on, arthroplasty has known considerable evolution, but UHMWPE remains the gold standard for artificial hips and now other artificial joints, including the knee and shoulder [1].

Despite a relatively successful history, the steadily increasing number of yearly procedures [2,3] and, above all, the dramatic increase of demand in younger, more active patients [4] have stimulated a constant research for optimized material formulations and processing procedures, to ensure a high level of performance and durability.

Each potential innovation has been accompanied by a great deal of pre-clinical trials, performed by researchers all over the world, often with very different methods and sometimes with contradictory results.

Only some of these studies were aimed at establishing a correlation between the chemical and morphological characteristics of the polymer and its mechanical properties and wear resistance. In some cases, retrieval studies have correlated the material properties to the clinical outcome of the implant.

The present work aims at exploring such a correlation through an analysis of the relevant literature that has appeared in the last decades.

## 2. UHMWPE

UHMWPE is a particular type of polyethylene (PE), with an exceptionally high molecular mass. The international Standards Organization (ISO 11542) (ISO, 2001) defines UHMWPE as having a molecular weight of at least 1 million g/mol, while the American Society for Testing and Materials (ASTM) specifies that UHMWPE has a molecular weight greater than 3.1 million g/mol [5]. Besides the molecular mass, the microstructure of the polymer also plays an important role in determining its physical, chemical and mechanical properties. UHMWPE, as most polyethylenes, is a semi-crystalline polymer composed of at least two interpenetrating phases: a crystalline phase, in which the macromolecules fold into ordered, crystalline lamellae and an amorphous, disordered phase, possibly intercalated by a partially ordered, so called all-trans, interphase [6,7].

The UHMWPE used in orthopaedic applications typically has a molecular weight between 3.5–6 million [8] and semi-finished bars and rods have a degree of crystallinity ranging around 50–55%. Such a precise combination of chemical structure, molecular mass and microstructure is at the basis of the peculiar balance of high mechanical properties and wear resistance that has made UHMWPE the material of choice in arthroplasty. A high entanglement density is associated with the ultra-high molecular weight; entanglements behave like physical crosslinks, which affect its crystalline morphology when the polymer is melt-crystallized. Unlike its lower molecular weight counterparts, UHMWPE crystallizes into a non-spherulitic structure comprising tortuous, defective lamellae. The high entanglement density is responsible for the relatively low crystallinity, compared to medium and low molecular weight, linear, high density polyethylene (HDPE) which can be melt crystallized to a degree of crystallinity of 70–80%. It is generally believed that the higher toughness and wear resistance of UHMWPE is associated with a large number of inter-lamellar tie molecules connecting adjacent lamellae, compared to HDPE. Furthermore, inter-spherulitic boundaries of HDPE are known to be weak since melt crystallization “sweeps” any impurities present in the melt to spherulitic boundaries which are also thought to contain a larger concentration of chain ends than the amorphous layers between lamellae. The suppression of spherulite formation in UHMWPE then must also contribute to the overall toughness and high wear resistance of UHMWPE. UHMWPE resin has an essentially zero melt flow index. As a result, upon melting of UHMWPE resin particles, that are usually approximately 50–150 μm in diameter, do not flow and instead retain their shape. This makes it extremely difficult to injection mold the powder to directly constitute implants and instead, the resin is compression molded or ram extruded into bars at elevated pressure and temperature, and then annealed to remove residual stresses. It is then machined into implants, packaged and sterilized prior to implantation.

It is quite clear that any processing step must be carefully controlled, from consolidation of the powder at elevated temperatures and pressures, to radiation sterilization, to addition of antioxidants or anti-slip agents or to any other specific treatment that can potentially influence such a balance and thus lead to changes in macroscopic mechanical and tribological properties As with any semicrystalline polymer, the microstructure and nanoscale lamellar morphology of UHMWPE depends on its thermal history during molding or ram extrusion. Unlike PEs with lower molecular weight, the consolidation of UHMWPE resin particles is a slow process due to the large time scales associated with reptation of chains of high molecular weight across resin boundaries to entangle with chains in adjacent resin particles and co-crystallize to form a fused article, assisted by pressure and temperature. Chain reptation, derived from the word “reptile”, is the thermal motion of entangled macromolecules in a tube, much like the slithering of snakes. In fact, it has been demonstrated that the extended-chain morphology of the UHMWPE resin upon melting leads to “chain-explosion” which is the dominant transport mechanism that leads to cocrystallization of chains across resin boundaries to fuse the resin, compared to the much slower chain reptation process [9]. The difficulty in fusion of resin particles makes the inter-powder boundaries weaker than the interior of resin particles, but overall provides a tough, highly wear resistant material with a relatively successful history of implantation as joint replacement devices.

## 3. “Historical” and Conventional Radiation Sterilized Polyethylenes

The term “historical” often identified polyethylenes that were sterilized by 25–40 kGy of gamma radiation in air [10]. These types of polyethylene have a long clinical history, starting from the first pioneering implants in the 1960s, up to the end of the 1990s, by which time most manufacturers had switched to inert-sterilized, barrier packaging PE and/or to crossliked PE. However, examples of gamma air sterilized polyethylenes can also sporadically be found in contemporary clinical applications [10,11].

**Figure 1:**
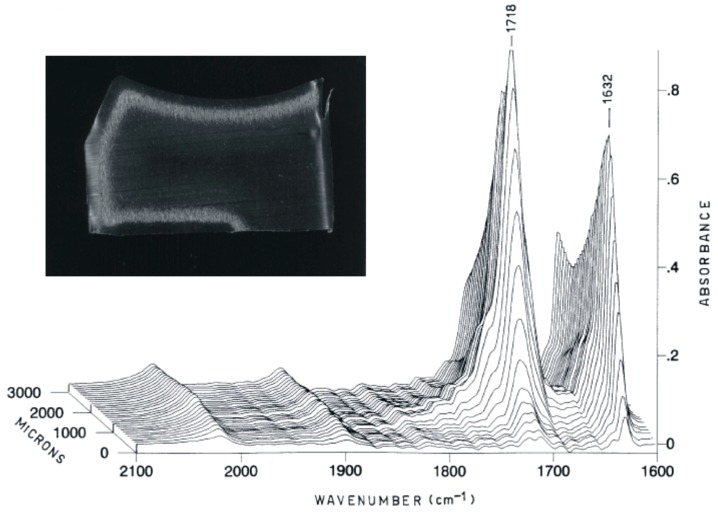
Cross-section of a gamma-air sterilized tibial insert, exhibiting a characteristic “crown-effect” (subsurface white band), along with its Fourier Transform InfraRed (FTIR) spectra, showing the presence of abundant oxidation products. Adapted from [17], with permission.

A body of literature, in particular across the late 1980s to the early 2000s, investigated the effects of radiation sterilization in air on the chemical, physical, mechanical and tribological properties of polyethylene [12,13,14,15,16,17,18]. Basically, high energy irradiation leads to the cleavage of chemical bonds of the PE chains, creating free radicals. The free radicals are highly reactive species that tend to produce a complex series of reactions, the extent of which is strongly dependent on the surrounding species available for reaction [19]. In inert atmosphere, the predominating effect of irradiation is the formation of crosslinks among the polymer chains. Conversely, in an air environment, the radicals can easily react with oxygen, triggering a cyclic, auto-sustained process that results in the formation of oxidation products on the backbone of the polymer and, more importantly, in predominating chain scissions, with a consequent overall decrease in its molecular mass, and significant changes to its morphology (Figure 1). In particular, it has been demonstrated that, immediately after irradiation in air, crosslinking and an increase in crystallinity are the dominant processes. With time, chain scission induced by oxidation prevails, resulting in a further increase in crystallinity [20,21,22], likely through the formation of a new phase of thinner crystallites in the amorphous region [13].

The extent and rate of radiation-induced oxidation depends on several factors, including the total absorbed dose and dose rate, the temperature of the sterilization facility, the oxygen availability and the sample thickness, which in turn governs the oxygen concentration distribution through the thickness of the implant. In addition, the oxidative process initiated during sterilization can continue, with variable yet low rates, during shelf storage and implantation (post-irradiation aging). Again, the rate and extent of oxidative degradation depend on the shelf-aging time and temperature and on the amount of available oxygen in the shelf and in vivo [19]. Further, it appears that mechanical stresses developed during in vivo use can also facilitate the oxidative process [23,24]. In summary, it follows that sterilization by high energy radiation in the presence of air can result in highly variable oxidation levels in polyethylenes, influenced by multiple factors.

**Figure 2:**
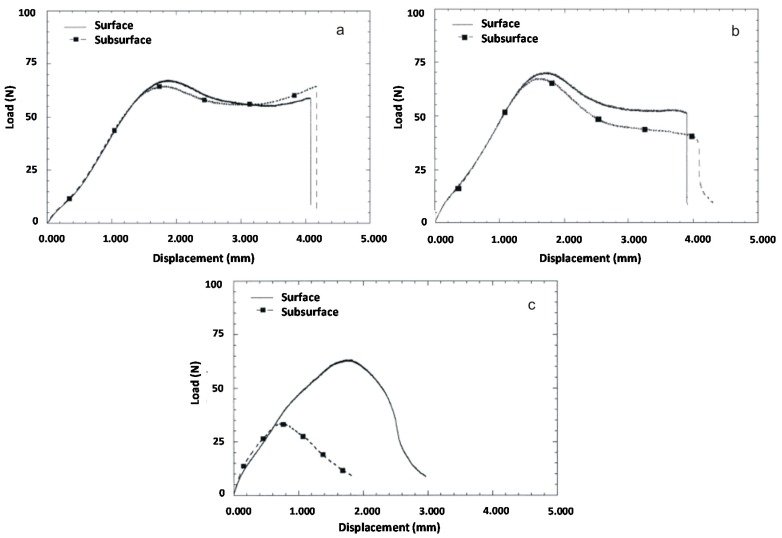
Small punch load displacement curves for gamma-air sterilized ultra-high molecular weight polyethylene (UHMWPE) tibial inserts at surface and subsurface locations: (**a**) control, unaged; (**b**) shelf-aged for 5 years; (**c**) shelf aged for 10 years. Adapted from [27], with permission.

**Figure 3:**
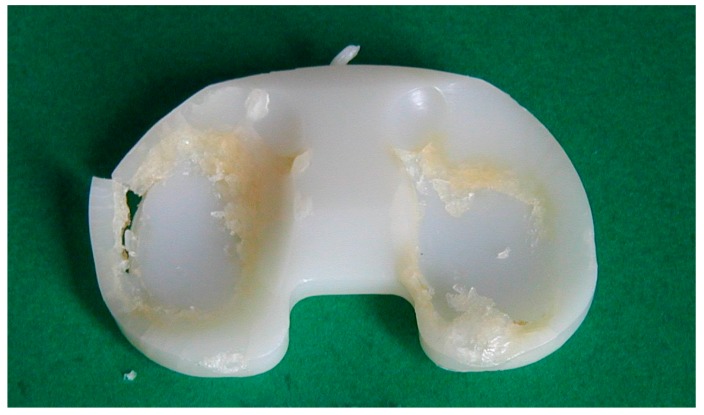
Retrieved polyethylene tibial insert showing severe wear damage, including severe delamination and wear (10 years in vivo).

Overall, oxidative degradation has been demonstrated to lead to significant changes in the mechanical properties of UHMWPE and, in particular, to embrittlement. Brittleness of polymers is well known to be correlated to the tensile properties [25,26]. Accordingly, an increase in elastic modulus and a decrease in the elongation to failure, ultimate stress and toughness has been demonstrated by a number of studies [27,28,29] (Figure 2); moreover, a decrease in fatigue crack propagation resistance has also been observed [20,30], while an often dramatic decrease in wear resistance (Figure 3) has been demonstrated by multiple in vitro and retrieval studies [22,29,31,32,33,34].

Wear in this material has typically been measured as weight loss per million cycles after accounting for absorption of bovine serum during articulation against a metal or ceramic counterface. A control specimen is typically loaded and soaked in bovine serum but not articulated and fluid absorption is measured periodically along with worn specimens. Wear rate has also been reported as wear factor, which is the weight loss normalized by load and the total wear path traveled [35,36]. 

It is worth mentioning though, that several in vitro studies reported markedly better wear performances of gamma-air irradiated polyethylenes vs. unirradiated ones. For example, Essner and coworkers [37], in a comprehensive study investigating the clinical relevance of hip simulator experiments, demonstrated that the wear volume of non-sterilized and EtO sterilized cups was twice that of gamma-irradiated in air cups. Similarly, Affatato et al. [38] showed that after 5M cycles in a hip simulator, EtO-sterilized specimens wore 1.2 times faster than those gamma-irradiated and the same result was confirmed even in a subsequent test in a regime of third-body wear [39]. McKellop et al. [40], in another hip simulator experiment, also found indistinguishable wear rates for two cups, both gamma-irradiated in air, made of different resins (GUR 4150 and 1020, with and without calcium stearate), and a 54% higher wear rate for a cup made with the same GUR 4150 resin, sterilized with ethylene oxide.

This likely occurs because, as highlighted above, gamma irradiation results in a combination of crosslinking and chain scission, the latter prevailing only after an extended time of post irradiation aging. Given the opposite effect of these two phenomena on the wear resistance of UHMWPE [17,33,41,42], it becomes apparent that radiation sterilized samples will exhibit less wear of unirradiated or EtO/gas plasma sterilized ones, at a short post irradiation time scale and storage conditions that allow crosslinking to prevail to a larger extent than chain scission. On the contrary, after suitable accelerated ageing or longer real time aging, in the shelf or in vivo, the wear and fatigue performances of gamma irradiated samples deteriorate considerably as a consequence of oxidative degradation and negate any short term benefits of crosslinking associated with sterilization methods that use ionizing radiation [21,29,32,33,43].

This observation prompted researchers in the field to implement strategies to take advantage of the benefit of the crosslinking induced by radiation treatments, yet minimizing the drawback of long-term oxidation.

The first adopted measure was to sterilize UHMWPE with high energy radiation in a low-oxygen environment (vacuum or inert gas, i.e., argon or nitrogen) [10,44,45,46]. This practice avoids contact with oxygen during sterilization and, if the liner is wrapped in a suitable barrier packaging, during the following shelf life as well [11,47]. Unfortunately, it does not prevent contact with the oxygen solubilized into polyethylene before packaging in the low-oxygen environment, nor with that available in vivo [43], so that some oxidation has also been observed in these polyethylenes, even if to much lower levels than for those radiation sterilized in air [11,48,49].

## 4. 1st Generation Highly Crosslinked Polyethylene

### 4.1. Improving the Wear Resistance

By the late 1990s, a large number of laboratory and clinical studies indicated that crosslinking provides substantial improvement in the wear resistance of UHMWPE. The mechanisms by which this improvement occurs were elucidated by various researchers [41,42,50,51,52]. Basically, wear of UHMWPE is believed to take place via plastic deformation of the polymer, with molecular alignment in the direction of motion that results in the formation of fine, drawn-out fibrils oriented parallel to each other. As a result of this arrangement, the UHMWPE wear surface may strengthen along the direction of sliding, while it weakens in the transverse direction. Wang et al. [50] concluded that, under the conditions of multi-directional motion, which may apply to both the hip and the knee joint, this orientation-softening phenomenon is predominantly responsible for the detachment of fibrous wear debris from the worn surfaces that have been observed in many reports [53,54,55]. Therefore, it has been postulated that, since crosslinking induces carbon-carbon bonds between adjacent chains, hereby reducing the chain mobility and inhibiting such molecular orientation, it would have been efficient in slowing down the formation of surface fibrils and rendering the polyethylene more resistant to wear [41,51,56].

**Figure 4:**
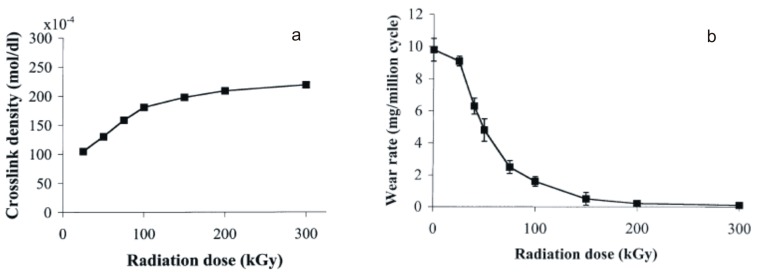
(**a**) Crosslink density and (**b**) pin-on-disk (POD) wear rate of irradiated UHMWPE as a function of increasing dose. Adapted from [42] with permission.

Although some controversies do exist in the literature, regarding the chemical mechanisms of radiation crosslinking of UHMWPE [19,56,57,58], most authors agree that the crosslinking density increases linearly up to radiation doses in the order of 100 kGy, above which it tends to a plateau (Figure 4a) [42]. However, the decrease in the tensile and fracture toughness continues at a radiation dose higher than 100 kGy [59,60]. Therefore, most of the “1st generation” highly crosslinked polyethylenes appeared in experimental in vitro and clinical studies at the end of the 1990s and in the early 2000s were irradiated to doses between 50 and 105 kGy [5].

### 4.2. How to Prevent Oxidation

However, as mentioned in the previous paragraph, numerous studies had already demonstrated an unacceptably low stability to oxidation of irradiated polyethylene. As a consequence, stabilization strategies were developed in order to minimize post-irradiation oxidative ageing. Basically, two different strategies were adopted: one involved post-irradiation melting of the polyethylene (remelting) [41,56], while the other included a thermal treatment, as well, but at a temperature below complete melting of the crystallites (annealing) [61,62]. In both cases, the rationale for the protocol was to eliminate or reduce the residual radicals, trapped in the crystalline phase of the polymer, as to avoid the oxidation cascade.

### 4.3. Microstructure, Mechanical Properties and Wear

Irradiation of polyethylene results in a slight increase in crystallinity with increases in the radiation dose, along with a marked decrease in elongation to failure and impact strength, a moderate decrease in ultimate strength and a slight increase in yield strength [41,63]. Post-irradiation remelting causes a significant decrease in crystallinity, accompanied by a reduction in yield and ultimate strengths [61,63] This likely occurs because the kinetics of crystallization is affected by the formation of a network structure due to the presence of crosslinks, which reduce the chain mobility and makes it more difficult for PE macromolecules to reptate and enter into growing lamellae. As a consequence, recrystallization of a radiation crosslinked UHMWPE always results in decreases in crystallinity [56,64]. Such a decrease in crystallinity is also associated to a decrease in fatigue resistance (Table 1) [30,60,63,65,66,67]. On the contrary, post-irradiation annealing does not cause a reduction in crystallinity, thus preserving superior yield, ultimate and fatigue properties, with respect to remelting [61,65,66,67], at least in the short term.

Both highly crosslinked formulations demonstrated superior wear resistance during in vitro tests, when compared to unirradiated or conventional gamma air/gamma inert sterilized polyethylenes.

For example, the wear rates measured using a bi-directional pin-on-disk (POD) on a set of polyethylenes, e-beam irradiated at room temperature in air to doses ranging from 25 to 300 kGy and subsequently melted (150 °C for 2 h under vacuum), were found to decrease sharply with increasing the radiation dose: from 9.6 ± 0.7 g/million cycles for the control, unirradiated sample, to 1.6 ± 0.3 g/million cycles for the 100 kGy irradiated and remelted sample, approaching an undetectable wear rate for radiation doses of the order of 300 kGy (Figure 4b) [42].

Similarly, acetabular cups machined from a polyethylene gamma-irradiated in air at doses ranging from 33 to 1000 kGy, remelted and EtO sterilized, demonstrated an 87% wear reduction with increase in the radiation dose from 33 to 95 kGy, when tested for 5 million cycles in a hip simulator; again, the wear decreased to undetectable levels at a radiation dose higher than 200 kGy [41]. In the same work, by observing the trade-off between the reduction in tensile properties and the increase in wear resistance with increasing the radiation dose, the authors concluded that approximately 100 kGy represents the most appropriate dose to reduce the wear below the threshold for clinically significant effects, simultaneously preserving sufficient tensile properties. 

Kurtz et al. [61] summarized the wear test results of seven independent hip simulator studies, all using the same hip simulator design and comparable conditions, of a conventional, gamma inert sterilized polyethylene (30 kGy gamma radiation, in nitrogen: N2-Vac™ Stryker Howmedica Osteonics, Mahwah, NJ, USA) vs. a highly crosslinked and annealed UHMWPE (75 kGy gamma radiation, annealing at 130 °C, 30 kGy gamma radiation in nitrogen for sterilization purpose, Crossfire™, Stryker Howmedica Osteonics, Mahwah, NJ, USA). Counterfaces included CoCr, alumina, and Zirconia ceramic femoral heads with sizes ranging between 28 and 36 mm. The linear wear rate was calculated in the first 2 to 3 million cycles of the test to facilitate comparison between studies. Despite a relatively broad distribution in wear rates, which may be partly explained by variations in liner design, femoral head material, and femoral head size, the survey demonstrated a 92% reduction in median wear rate for the highly crosslinked and annealed UHMWPE, relative to the conventional, gamma inert sterilized polyethylene.

### 4.4. Does the Counterface Matter with Crosslinked Polyethylene?

The influence of the type of counterface on the wear behavior of crosslinked polyethylene was also investigated. Bistolfi and coworkers [64] showed that a moderate dose (50 kGy) of radiation crosslinking increased the wear resistance of UHMWPE against a CoCr counterface in a POD apparatus 7-fold, but the coupling of remelted, crosslinked UHMWPE against a smoother alumina counterface led to a 20-fold increase in wear resistance. Affatato et al. [68] tested e-beam highly crosslinked and remelted hip liners (95 kGy at 40 °C, thermally treated at 150 °C for 6 h, Gas Plasma sterilized. Longevity™, Zimmer Inc., Warsaw, IN, USA) in a hip simulator for 3 M cycles, against deliberately scratched femoral heads (mean surface roughness, Ra: 0.12–0.14 μm). Even in such demanding conditions, they reported a 40 times lower wear rate for the crosslinked material than for conventional, gamma-nitrogen sterilized UHMWPE.

A previous study [36] also demonstrated that moderately crosslinked UHMWPE (40 kGy in vacuum, no thermal treatment) exhibited a 30% reduction in wear rate compared to unirradiated polyethylene, when articulating on smooth CoCr femoral heads. However, upon intentionally scratched CoCr femoral heads the wear rate was found to be higher for the moderately crosslinked polyethylene than for the non-crosslinked materials. Most importantly, the moderately crosslinked polyethylene generated a higher percentage volume of smaller, more biologically active particles, thus resulting in a similar index of functional biological activity. The authors concluded that this evidence provides a clear message that is preferable to use crosslinked materials against damage-resistant ceramic heads, to prevent the possibility of wear acceleration owing to third body damage to metallic femoral heads. Another study by the same group [69] demonstrated a fivefold lower wear for 75 and 100 kGy gamma irradiated and remelted polyethylenes, compared to control unirradiated or 25 kGy irradiated in air, when tested for 5 M cycles in a hip simulator, against CoCr femoral heads. The wear reduction was found to be significantly higher for the material irradiated to 100 kGy. In this experiment, a large reduction in the functional biological activity of the wear particles generated by the highly crosslinked material was also observed and it was attributed to the overall lower wear volume found with this material. The same study also highlighted the influence of “bedding-in”, due to creep deformation, on the greater wear volume observed in the first million cycles for all polyethylene configurations.

### 4.5. Thermal Treatments and Oxidation Stability

Regarding the oxidation stability of the two formulations of 1st generation highly crosslinked polyethylenes, McKellop and coworkers compared the oxidation stability and wear resistance of the same set of samples mentioned above [41], with or without remelting, following accelerated ageing at 80 °C in air for 20 to 30 days. The remelted cups exhibited little or no oxidation after artificial aging and no white bands, that are generally interpreted as a visual indication of oxidation [17], were present on the cross sections of the remelted cups. In contrast, without remelting, there was considerable oxidation of the crosslinked cups, with subsurface oxidation peaks. White bands were present on the cross sections at the levels corresponding to maximum oxidation. Despite this oxidation, the wear rates of the cups, with or without remelting, were comparable before and after aging. However, it was observed that, being that the oxidized zones were located about 0.5 mm below the surface and given the low wear rates of the crosslinked cups, the wear did not penetrate into the oxidized layers of the not remelted cups, under the conditions of the experiment, but that, likely, this subsurface oxidation could cause an increase in the wear rate after extended clinical use. This observation prompted the authors to postulate that remelting is an essential step to optimize the long-term performance of crosslinked cups.

Muratoglu and coworkers [70] tested the oxidative stability of two highly crosslinked polyethylenes, one of which had been ebeam irradiated to 100 kGy, remelted (150 °C, 2 h) and terminally gas plasma sterilized, while the other had been gamma irradiated to 75 kGy, annealed below the melting temperature (120 °C) and gamma sterilized (25–35 kGy) in nitrogen. The POD wear rates of the two unaged samples were comparable and much lower than those of conventional, uncrosslinked samples. Residual free radicals were detected in the annealed samples by Electron Spin Resonance (ESR), while they were not found in the remelted samples. Following accelerated ageing in air at 80 °C for 3 weeks, the annealed samples developed significant oxidation and their wear rate was found to increase by over an order of magnitude, while no oxidation or significant changes in the wear rate were observed for the remelted formulation. 

**Figure 5:**
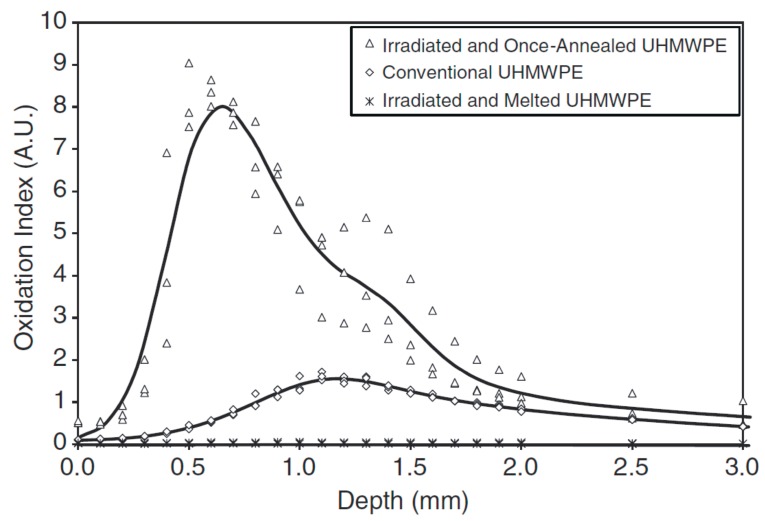
Oxidation profiles of conventional UHMWPE (gamma-inert sterilized, 25–40 kGy), irradiated and once-annealed UHMWPE (CrossfireTM Stryker-Howmedica-Osteonics; Rutherford, NJ, USA), and irradiated and melted UHMWPE (DurasulTM Zimmer, formerly Centerpulse; Austin, TX, USA) after 128 weeks of real-time aqueous aging at 40 °C. Reproduced with permission from [71].

In another study [71], the same annealed formulation was compared to a warm irradiated (95 kGy at 120 °C) and remelted PE. The two samples were real-time aged, in a pure, distilled water bath at 40 °C for 128 weeks. Again, at increasing aging times, the annealed sample showed increasing oxidation, with a maximum located at a subsurface level, while no oxidation was detected in the remelted PE (Figure 5). Upon testing the hip simulator wear rate of the real-time aged, annealed components, the authors also observed that it was higher than the literature reported values of other contemporary highly crosslinked UHMWPEs.

Collier et al. in an extensive study that compared the physical and mechanical properties of commercial, 1st generation crosslinked polyethylenes from six orthopaedic manufacturers [72], also concluded that, again, crosslinked/annealed PE was prone to oxidation, following accelerated aging, while all of the tested crosslinked/remelted materials showed oxidation resistance equal to that of never-irradiated polyethylene before and after accelerated aging and thus have the potential to remain relatively unaffected by oxidation throughout their duration in vivo. 

Most of the aforementioned studies attributed the differences in the oxidative stability between remelted and annealed crosslinked polyethylenes to that a thermal treatment above the melting temperature (remelting), leads to a complete melting of the polymer crystallites, hereby allowing termination of the radicals trapped in the crystalline phase and thus brings the amount of residual radicals to undetectable levels [71,73]. Conversely, annealing just below the melting temperature leaves a measurable amount of free radicals in the polymer matrix, that can react with oxygen over time, thus triggering the oxidation cascade [61].

### 4.6. Clinical Outcomes and Retrieval Studies

Several clinical studies have reported superior in vivo wear performance of highly crosslinked PE, compared to conventional polyethylene in hip replacements [74,75,76,77,78,79,80,81,82,83,84,85,86,87], most of which were included in an extensive, systematic review of the clinical outcomes of 1st generation crosslinked polyethylene appearing at the beginning of the present decade [88]. Conversely, very few studies exist on the clinical performances of highly crosslinked polyethylene in the knee [89,90,91], partly because of a more limited introduction of this material in knee arthroplasty, due to recurrent concerns on its suitability, as a result of the loss in mechanical properties with increasing doses of radiation [88] and partly because of the lack of validated methods of determining the wear and damage rate of these components in vivo [56].

A number of retrieval studies have also reported on the chemical, physical and mechanical properties of these highly crosslinked PE following in vivo aging. According to predictions of in vitro studies, oxidation of the highly crosslinked/annealed formulation was reported by a number of authors [92,93,94,95]. Highly variable oxidation levels were measured in all the retrieval groups included in these studies, although none of the components had been explanted specifically for polyethylene wear. All studies agreed that the highest oxidation is generally measured at the superior rim of the component and not at the bearing surfaces and it was postulated that this occurs because of the greater exposure of the rim area, compared to the bearing area, to molecular oxygen in the in vivo environment [96].

On the other hand, failures of some specific designs of highly crosslinked/remelted have also been described in the literature [97,98]. Rim fractures have been observed in some components and have been attributed to the combination of reduced fatigue resistance induced by irradiation and remelting, with malpositioning and with particularly demanding designs, in terms of stress concentration [97].

As already mentioned, crosslinked/remelted polyethylenes did not contain free radicals at the time of implantation and were expected to be stable to oxidation. Unexpectedly, some recent literature studies reported that measurable levels of oxidation were observed in some retrievals [99,100,101,102,103,104]. Two concurrent causes have been tentatively identified to explain such a surprising phenomenon. One is that the cyclic loading to which the components are exposed during in vivo service might have generated free radicals, initiating mechano-oxidation [24,102]. The other involves the lipids absorbed in vivo from the synovial fluid [105]. Oral et al. [106] demonstrated that, under accelerated aging conditions, squalene, a precursor in cholesterol synthesis, has the potential to accelerate oxidation in radical-free, irradiated and melted UHMWPE. 

Although the clinical significance of such adverse behaviors of the 1st generation highly crosslinked PE is still under debate, their occurrence stimulated the research of alternative polyethylene formulations to overcome the observed drawbacks.

## 5. 2nd Generation Highly Crosslinked Polyethylene

### 5.1. The Need for a 2nd Generation Crosslinked Polyethylene

The rationale at the basis of a new generation of highly crosslinked polyethylene was to maintain the superior wear resistance demonstrated by the 1st generation crosslinked PE, while also retaining the mechanical properties and fatigue resistance of the uncrosslinked material and ensuring stable properties, thus oxidative stability, over time.

### 5.2. Sequentially Irradiated and Annealed Highly Crosslinked Polyethylene

As discussed in the previous paragraph, annealing below the melting temperature preserves most of the original UHMWPE microstructure. Highly crosslinked/annealed PE had been demonstrated to retain mechanical properties and fatigue resistance similar to conventional, gamma-nitrogen sterilized (but unoxidized) material; however, the presence of free radicals in the latter leaves the polyethylene exposed to oxidation [62]. Dumbleton and coworkers [107] proposed that it might have been possible to create a highly crosslinked PE by using a sequential irradiation and annealing process. Since a high (≥100 kGy) radiation dose creates many crosslinks that reduce the chain mobility, preventing an efficient elimination of the free radicals by annealing, they postulated that by using a low radiation dose (30 kGy), hereby spacing the crosslinks wider and providing higher chain mobility, annealing would have been more efficient in eliminating free radicals. High crosslinking levels could still be obtained by sequentially repeating the irradiation and annealing steps so that the polyethylene cumulatively receives a high dose of radiation (approx. 90 kGy).

This 2nd generation crosslinked PE (X3™, Stryker Orthopaedics, Mahwah, NJ, USA) was terminally sterilized with gas plasma, so as to avoid the introduction of more free radicals with sterilization. Literature studies reported similar or only slightly reduced mechanical and fatigue properties compared to conventional PE [62,67,107,108] and superior wear resistance in vitro [67,107,108,109] and in vivo [110,111]. However, incomplete melting following irradiation leaves a low but measurable amount of free radicals in the material [107,109], that was demonstrated to oxidize in vivo [100,103,112,113,114]. Although most studies agreed that the measured levels of oxidation were generally low, in particular much lower than those reported for the 1st generation once-annealed highly crosslinked PE [100], and were not associated with a decline in the clinical performance of the sequentially annealed PE [113], observations of pitting and substantial material loss as well as subsurface white banding and cracking were also reported in a knee retrievals study [114]. More investigations and longer follow-up are therefore necessary to assess the overall in vivo performance of this type of 2nd generation highly crosslinked PE.

### 5.3. Antioxidants: The New frontier

An alternative approach to the 2nd generation highly crosslinked polyethylene involves the addition of an anti-oxidant stabilizer, in order to efficiently inhibit the oxidative degradation, without the need for a thermal treatment of the irradiated polyethylene, so as to preserve the original morphology, mechanical properties and fatigue resistance [115]. Although this is a very common approach to stabilize polyolefins against oxidation [116], the addiction of additives to biomedical UHMWPE had created concerns related to their biocompatibility for a long time. The first scientific papers and patents mentioning the possibility of using vitamin E (α-tocopherol) as a biocompatible stabilizer for UHMWPE [18,117] and exploring the subject are dated back to the late 1990s and onwards [118,119,120], but the first vitamin E-stabilized UHMWPE did not appear on the orthopedic market before 2007 [121,122]. Since then, though, a number of studies have investigated the performances of crosslinked UHMWPE incorporating vitamin E [115,121,122,123], at the moment by far the most used, and other stabilizers [124,125,126,127,128].

Two methods are currently used to incorporate vitamin E into UHMWPE [5,115]. One involves blending of vitamin E with UHMWPE powder before consolidation and radiation crosslinking [121]. The presence of vitamin E, a radical scavenger, in UHMWPE during irradiation protects the polymer from oxidation [118,129,130,131] but reduces the efficiency of crosslinking [120,132]; therefore, the balance between the vitamin E concentration and the radiation dose must be optimized to obtain a simultaneously wear- and oxidation-resistant UHMWPE [133]. The alternative method is the diffusion of vitamin E into UHMWPE after radiation crosslinking [122]. The crosslinking efficiency of UHMWPE is not adversely affected by this method, but a homogenization step is required after incorporation to obtain adequate antioxidant concentration throughout the implants [134].

### 5.4. Mechanical Properties, Oxidation Stability and Wear Performances

Several studies have investigated the mechanical properties of both formulations of vitamin E stabilized PE. No significant differences in the static mechanical properties, nor in the fatigue crack propagation resistance have been observed when comparing virgin UHMWPE with blends containing up to 5000 ppm of vitamin E [118,135]. Conversely, it has been demonstrated that highly crosslinked vitamin E-stabilized UHMWPE exhibits improved material properties compared to 1st generation highly crosslinked PE. For example, vitamin E-doped 100-kGy irradiated UHMWPE demonstrated a 58% improvement in fatigue strength compared to irradiated and melted UHMWPE [136], attributed to the avoidance of the loss of crystallinity during post-irradiation melting. Moreover, the ultimate strength, yield strength, elongation at break, and fatigue resistance of crosslinked vitamin E-stabilized UHMWPE were significantly higher than that of 100 kGy–irradiated and melted UHMWPE [137].

**Figure 6:**
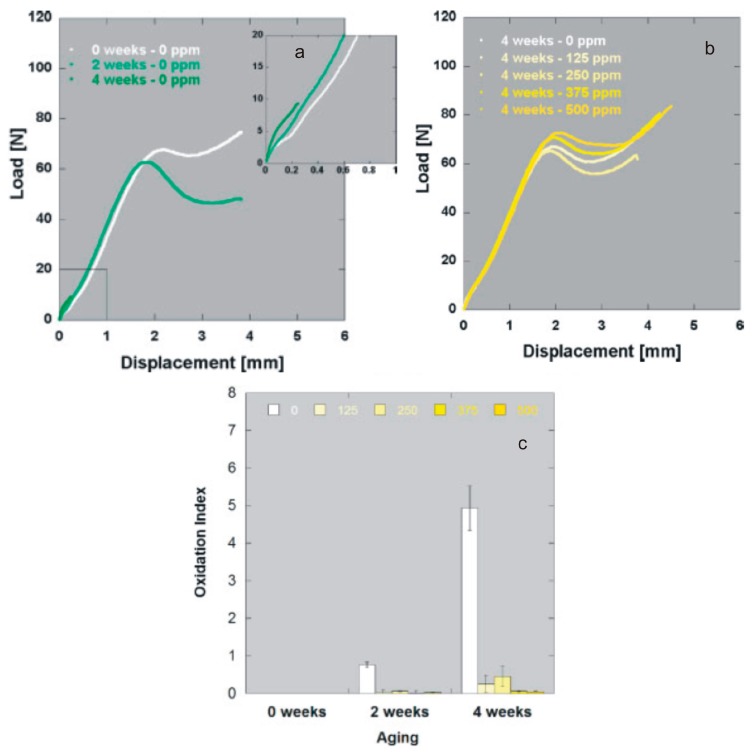
(**a**) Small punch curves of virgin UHMWPE irradiated at 75 kGy in air: control and aged 2/4 weeks (ASTM F2003-00); (**b**) Small punch curves of UHMWPE blended with increasing concentrations (0–500 ppm) of vitamin E, irradiated at 75 kGy in air and aged 4 weeks; (**c**) Oxidation indexes of UHMWPE blended with increasing concentrations of vitamin E (0–500 ppm), irradiated at 75 kGy in air and aged 2/4 weeks. Adapted from [133], with permission.

In addition, Vitamin E-stabilized PE has demonstrated superior oxidation stability under a variety of accelerated aging conditions. For example, blends of UHMWPE with 500, 1000 and 5000 ppm of vitamin E, gamma irradiated to doses up to 100 kGy were found to be more oxidatively stable than virgin, unirradiated UHMWPE, following accelerated aging at 90 °C in air [129]. Oral et al. [136] showed that vitamin E-doped, irradiated UHMWPEs developed significantly lower oxidation levels compared to 100-kGy irradiated UHMWPE after 5 weeks of accelerated aging at 80 °C in air, while Kurtz and coworkers [133] found that blending PE with doses of vitamin E as low as 125 ppm was sufficient to avoid oxidation and maintain baseline mechanical and chemical properties, after irradiation up to 75 kGy, through two weeks of accelerated aging, according to ASTM F 2003 (i.e., 70 °C and 5 atm oxygen) (Figure 6).

The wear resistance of this 2nd generation highly crosslinked formulation has been extensively tested in several in vitro experiments, under a variety of challenging conditions, and has been compared to conventional and 1st generation highly crosslinked formulations before and after both accelerated and shelf-aging. 

In a POD wear test, highly crosslinked Vitamin E-doped PE exhibited comparable wear rates to 1st generation highly crosslinked PE irradiated to the same dose [136], while it demonstrated a 4-fold to 10-fold decrease, respectively, from that of conventional UHMWPE in a hip simulator experiment with and without the addition of third-body particles [137].

Bellare et al. [138] compared the wear rates of vitamin E blended (1000 and 5000 ppm) vs. virgin UHMWPE, gamma irradiated to 30 and 100 kGy, before and after shelf aging for two years. The samples were also characterized for physical properties and crosslinking density: it was found that the crosslinking density increases with the radiation dose, but decreases with the vitamin E content. This was not surprising, as it is known that vitamin E, as a radical scavenger, can inhibit the crosslinking reactions [19]. Accordingly, the wear rates of the vitamin E containing samples were comparatively higher than that of virgin PE irradiated to the same dose, but lower than that of unirradiated PE. Conversely, after two years of shelf aging, virgin, irradiated, UHMWPE showed high oxidation levels, while vitamin E, as expected, proved to be very effective in retarding oxidation.

Similarly, while comparing the wear behavior of standard, unirradiated PE to that of two formulations of crosslinked polyethylene, with and without vitamin E (1000 ppm), both irradiated to 70 kGy, Affatato et al. [139] found that the vitamin E blended PE exhibited a much lower wear than conventional ultra-high molecular weight polyethylene, but wore more than the traditional crosslinked polyethylene. Again, this was correlated to the lower crosslinking density induced by the same radiation dose in the presence of vitamin E.

These observations suggest that, if vitamin E is necessary to avoid oxidation, then the radiation dose must be optimized in order to obtain enough crosslinking density to preserve the wear resistance of unstabilized, crosslinked PE.

Given the concerns mentioned above on the use of 1st generation highly crosslinked PE in knee arthroplasty, the vitamin E crosslinked formulations represented an attractive alternative, in particular in this application. For this reason, many experimental studies investigated the performance of vitamin E stabilized PE for use in the knee with promising results. For example, while comparing the wear rate of two designs (cruciate-retaining and posterior-stabilized) of highly crosslinked UHMWPE doped with vitamin E to that of γ-inert–sterilized direct compression-molded UHMWPE, Haider and coworkers [140] found that the former exhibited up to 86% reduction in wear for both designs.

In another knee simulator experiment, UHMWPE blended with 1000 ppm of vitamin E and radiation sterilized with 30 ± 2 kGy, was artificially aged according to ASTM F2003-2 and tested for 5 million cycle without showing structural failures, as conventional PE did under similar conditions [141]. Similarly, vitamin E-stabilized highly crosslinked components tested after accelerated aging, in comparison to standard, unaged, UHMWPE, demonstrated a 57% reduction in wear [142]. 

Teramura and coworkers [143] evaluated the wear behavior of direct compression molded tibial components, made of virgin and vitamin E blended (3000 ppm) UHMWPE. The components were gamma sterilized in air (ca. 25 kGy) and aged at 80 °C in air for 23 days. It was demonstrated that the wear behavior of the vitamin E-containing UHMWPE was not significantly affected by aging and showed a 12-fold reduction, compared to that of the aged virgin UHMWPE. The wear debris particles were also evaluated for shape and size and no significant differences were observed between the two materials.

### 5.5. The Effects of Vitamin E on UHMWPE Wear Debris

Bladen et al. [144] demonstrated, as well, that particles generated by UHMWPE in a pin-on-plate wear simulator with and without VE were not significantly different in size distribution. They also investigated the biological activity of particles containing doses of vitamin E up to 30,000 ppm, and found that the vitamin E-containing particles secreted much lower levels of osteolytic mediators, tumor necrosis factor-alpha and interleukin, than particles of virgin UHMWPE at comparable volume doses.

To investigate the effects of particulate debris in vivo, Bichara et al. [145] compared the effect of clinically-relevant sized particulate debris from a crosslinked vitamin E-doped (8000 ppm) vs. a crosslinked/remelted UHMWPE bearing component in a murine calvarial bone model. A statistically significant difference in the amount of fibrous tissue was observed between the two materials, with virgin UHMWPE wear particles inducing the greatest amount of inflammatory tissue. 

In addition, it has been shown that vitamin E may also exert some antibacterial activity [146,147,148]. This is likely due to differences in the surface hydrophilicity between virgin and vitamin E-stabilized polyethylene that in turn results in slightly different protein adhesion and biofilm formation.

### 5.6. Clinical Outcomes and Retrieval Studies

Literature reports on the clinical outcome of vitamin E stabilized PE are still quite limited in number, but their results are overall promising. 

One recent study [149] comparing vitamin E-blended crosslinked polyethylene and conventional gamma-inert sterilized at three years follow up, demonstrated that the femoral head penetration was significantly lower for vitamin E-blended HXLPE and similar to that reported for 1st generation HXLPE. Also, no specific complications related to the material were reported in the short-term.

Prospective studies at two to three years follow-up demonstrated no significant differences in femoral head penetration rates between vitamin E doped and virgin highly crosslinked liners [150,151,152].

However, one study reporting head penetration into vitamin E doped highly crosslinked liners at five years demonstrated less wear compared to that previously reported for 1st generation highly crosslinked PE, at the same interval of five years. Further, the study demonstrated that, after settling of the liners in the early period, no significant head penetration occurred from two- to five-year follow-up and the authors remarked that, overall, the wear observed in the study was well below that at which osteolysis becomes a serious concern [153].

Rowell et al. [154] analyzed 15 surgically retrieved vitamin E-stabilized crosslinked UHMWPE liners to assess their oxidative stability, extent of lipid absorption in vivo, free radical content, hydroperoxide index and extent of visible wear damage after in vivo service (0.1–36.6 months). Their results evidenced promising results, in terms of oxidative resistance and absence of significant surface damage, while also suggesting long-term stability through a reduction in free radical content over time and lack of oxidation after ex vivo aging in air. 

Similarly, Currier et al. [155] analyzed 25 antioxidant containing polyethylene tibial insert retrievals, including one formulation with an antioxidant other than vitamin E, with in vivo time of 0–3 years. All of the antioxidant materials appeared to be effective at minimizing oxidation over the in vivo period of the study. In addition, the authors speculated that the absence of subsurface peak oxidation suggest that antioxidant PE could be successful in preventing oxidation-mediated fatigue.

## 6. Conclusions

UHMWPE remains the most commonly used bearing material in total joint arthroplasty due to its long and relatively successful history. However, UHMWPE is a complex material and its mechanical and tribological behavior is strongly dependent on the morphology and chemical alterations induced by processing conditions, such as sterilization using ionizing radiation or crosslinking. The need for increasing the life expectancy of the implants, in order to meet the demand in younger and active patients, has led to a considerable evolution in the manufacturing of the UHMWPE orthopedic devices over the last few decades. From gamma-air sterilized “historical” polyethylenes, to the first and second generation of highly crosslinked materials, the wear resistance, mechanical properties and overall performances of the UHMWPE biomaterials have greatly improved, as has been extensively reported in the literature. Advances in the sterilization techniques have enabled a substantial reduction of the oxidative degradation experienced by “historical” implants, greatly lowering the incidence of wear-induced osteolysis and early mechanical failures. First generation crosslinked PE has further reduced wear, but at the expenses of fatigue resistance and/or oxidative stability, while 2nd generation crosslinked PE has been introduced to overcome the latter drawback and, overall, it has shown promising, yet early results, despite some issues regarding the oxidative stability of the sequentially irradiated and annealed PE.

Nevertheless, the clinical outcome of some of the newest formulations is still largely unexplored. It is therefore of paramount importance to ensure a close, continuous monitoring of the clinical performances of the contemporary UHMWPE biomaterials.

## Figures and Tables

**Table 1:** Physical properties and fatigue crack propagation data for a set of standard and crosslinked/remelted UHMWPE (GUR 1050), at increasing gamma radiation doses. All crosslinked samples were remelted at 170 °C for 4 h and subsequently annealed at 125 °C for 2 days. Adapted from [60] with permission.

	Control	50 kGy	100 kGy	200 kGy
**Crystallinity (%)**	50.1 ± 0.5	45.6 ± 0.7	46.3 ± 0.8	47.1 ± 0.4
**Lamellar thickness (nm)**	20.0	18.1	18.7	19.1
**Elastic modulus (MPa)**	495 ± 56	412 ± 50	386 ± 23	266 ± 30
**Yield stress (MPa)**	20.2 ± 1.0	19.9 ± 0.8	18.9 ± 0.7	20.2 ± 1.0
**True stress at break (MPa)**	315.5 ± 31.6	237.6 ± 12.3	185.7 ± 7.5	126.0 ± 14.0
**Decrease in true stress at break (%)**	-	24	41	60
**ΔK_incep_ (MPa√m)**	1.41	0.91	0.69	0.55
**Decrease in ΔK_incep_ (%)**	-	35	51	61
